# Chronic political instability and HIV/AIDS response in Guinea-Bissau: a qualitative study

**DOI:** 10.1186/s40249-021-00854-z

**Published:** 2021-05-11

**Authors:** Joshua Galjour, Philip J. Havik, Peter Aaby, Amabelia Rodrigues, Laura Hoemeke, Michael J. Deml, Jinkou Zhao, Emmanuel Kabengele Mpinga

**Affiliations:** 1 Institute of Global Health, University of Geneva, Geneva, Switzerland; 2 The Global Fund to Fight AIDS, Tuberculosis and Malaria, Geneva, Switzerland; 3 Centre for Global Health and Tropical Medicine (GHTM), Institute of Hygiene and Tropical Medicine, Universidade Nova de Lisboa, Lisbon, Portugal; 4 Bandim Health Project, Bissau, Guinea-Bissau; 5 Gillings School of Global Public Health, University of North Carolina, Chapel Hill, NC USA; 6 Institute of Sociological Research, Department of Sociology, University of Geneva, Geneva, Switzerland

**Keywords:** Guinea-Bissau, HIV/AIDS, Political instability, Governance, Global health, Qualitative health research

## Abstract

**Background:**

The Republic of Guinea-Bissau in West Africa has a high HIV/AIDS disease burden and has experienced political instability in the recent past. Our study used qualitative methods to better understand key stakeholders’ perceptions of the effects of chronic political instability on the HIV/AIDS response in Guinea-Bissau from 2000 to 2015 and lessons learned for overcoming them.

**Methods:**

Seventeen semi-structured in-depth key informant interviews were conducted in Bissau, Guinea-Bissau in 2018. Interviews were recorded and transcribed verbatim, coded thematically, and analyzed inductively.

**Results:**

Four themes emerged: (1) constantly start over; (2) the effects of instability rippling from central level throughout the health pyramid; (3) vulnerable populations becoming more vulnerable; and (4) coping mechanisms.

**Conclusions:**

Stakeholders from government, civil society, and donor organizations have recognized instability’s effects as a barrier to mounting an effective local response to HIV/AIDS in Guinea-Bissau. To mitigate the effects of the country’s political instability on the health sector, concerted efforts should be made to strengthen the capacities of health officials within the Ministry of Health to shield them from the effects of the country’s political instability.

**Graphic abstract:**

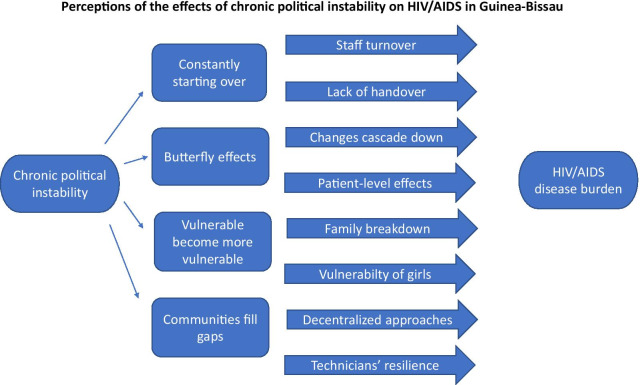

## Background

The Republic of Guinea-Bissau has experienced political instability in the recent past. According to the World Bank, Guinea-Bissau is tied with Somalia for the number of planned or successful coups recorded, symptomatic of “weak institutions, fragmented elites, and high rent seeking” [1]. Following a protracted struggle for independence against Portugal, Guinea-Bissau achieved independence in 1974. A nearly year-long civil war followed in 1998–1999 and its chaotic repercussions revealed social and political divisions throughout government and civil society [2]. The challenges posed by its unstable political order continue to the present, most recently following the disputed presidential election in December 2019 which led to two Presidents and two Prime Ministers concurrently holding office [3].

The HIV/AIDS epidemic in Guinea-Bissau may be considered an epidemiological outlier compared to neighboring countries. The country has a high HIV/AIDS prevalence (6.7%, 2016) and the world’s highest prevalence of HIV-2 (2.8%, 2016), although decreasing [4]. Its prevalence is considerably higher than in neighboring Senegal (0.4%), Gambia (1.9%) or Guinea (1.4%) [5]. Antiretroviral therapy (ART) coverage remains low at 33% (29–37%) [6], and the country is classified by World Health Organization (WHO) as one of the 30 high burden countries for tuberculosis/HIV co-infection [7]. This study will investigate if political instability has adversely affected the HIV/AIDS response.

Political instability may take the acute forms of armed conflict, violent regime change, or politically motivated assassinations. Ndokang and Tsambou [8] define government instability as “unforeseen and unexpected events such as the end of a government or of an electorate that occurs either legally or by force.” Political instability may also manifest itself in the state’s chronic inability to perform its basic functions over a protracted period. This form, oftentimes no less deleterious than sudden crises, suggests a governance deficit. If governance is defined as “solving problems and creating opportunities, and creating the structures and processes for doing so,” [9] then weak governance might be perceived through the breakdown of the state’s ability to address through non-violent political processes the challenges faced by its populace.

Political instability may be felt most profoundly in the state’s capacity to deliver social services, including in the health sector. According to the World Bank, “good governance in health systems promotes effective delivery of health services” [10]. The WHO defines governance “as a political process that involves balancing competing influences and demand" and includes leadership and governance as one of the six building blocks of well-functioning health systems [11]. One of the aspirations of the African Union’s Agenda 2063 is good governance on the African continent and one of its stated goals is “healthy and well-nourished citizens” [12].

Guinea-Bissau is a small, low-income country of approximately 1.9 million inhabitants and currently growing at a rate of 2.5% [13]. The population under 25 years is 62%, the total fertility rate is 4.5, and life expectancy at birth is 58 [13]. Its main export is the cashew nut, whilst its economy depends on subsistence farming and foreign aid [14, 15]. Narco-trafficking of drugs from South America to Europe is said to fuel the country’s instability and has led to the country having been labeled, problematically, as a narco-state [16]. The armed forces have played a disproportionately large role in state affairs and are viewed by many as having historically fueled the country’s chronic instability [17]. The planning, human resources and outcomes of an already underfunded and understaffed public health service [18] has been affected by chronic political instability, for example, through the proliferation of informal payments [19].

Indeed, the literature documents the diverse effects of political instability on the Bissau-Guinean health sector. For instance, the effects of the 1998–1999 war on health outcomes have been explored on HIV outcomes among police officers [20] and pregnant women [21] as well as the effects of irregular TB treatment during the civil war [22]. Russo et al. describe the country’s “stable political instability,” and to the “nature, persistence and evolution of its human resources for health crisis” [23]. In documenting the origins and performance of local health research, Kok et al. assert that the “post-colonial, volatile and resource-dependent context” has also influenced health systems research in Guinea-Bissau [24]. An article by Rasmussen et al. is based on a study that is rare in its attempt to empirically link specific health services or outcomes—such as HIV testing availability among pregnant women—to periods of political instability [25].

Our study employed qualitative methods to shed light on key stakeholders’ perceptions of the effects of chronic political instability on the HIV/AIDS response in Guinea-Bissau and lessons learned for overcoming these obstacles. A deeper qualitative understanding of the intertangled relationship between political instability and the HIV/AIDS response may offer insights about Guinea-Bissau and potentially similar contexts characterized by chronic political instability and may suggest new strategies for strengthening resilience.

## Methods

### Study design 

This study adopted an exploratory and qualitative design to understand stakeholders’ perceptions of the impact of chronic political instability on the HIV/AIDS in Guinea Bissau between 2000 and 2015.

### Participant selection

Eighteen key informants were purposively selected based on their roles, experiences, and knowledge of and role in the HIV/AIDS response in Guinea-Bissau during the years 2000 to 2015. The inclusion criteria followed were involvement in the HIV/AIDS response during the years 2000–2015, ability to speak English, French or Portuguese, and availability and willingness to provide informed consent to participate in the study. Eighteen key informants were purposively selected based on their roles, experiences, and knowledge of and role in the HIV/AIDS response in Guinea-Bissau during the years 2000–2015. The key informants played roles in the HIV response in the areas of policy, planning, implementation, or monitoring and evaluation during the Millennium Development Goals era (2000–2015). Key informants variously represented government (Ministry of Health, National AIDS Secretariat), civil society, United Nations agencies, researchers, and implementers. Some informants had been active in one or more of these areas in their professional careers. One of the 18 targeted key informants declined to participate in the study for reasons not stated. As a result, a total of seventeen key informants participated.

The selection of key informants was guided by the desire to recruit an appropriate mix of local and international actors, Ministry staff and development partners, and informants who had remained in Guinea-Bissau during the period and those who had spent at least part of this period outside Guinea-Bissau.

### Data collection

We used semi-structured qualitative key informant interviews to understand perceptions among key stakeholders of the effects of chronic political instability on the HIV/AIDS response in Guinea-Bissau during the years 2000–2015. An interview guide was developed based on firsthand experiences with Guinea-Bissau’s HIV/AIDS response and was used to orient the interviews. The printed guide in English and Portuguese was made available to the interviewees as a reference both before and during the interview. The semi-structured nature of the interviews allowed the interviewer to tailor the order of the questions and the use of probing questions to the specific role, background, or expertise of the key informant. Rather than systemically asking each question in the guide, the interview guide’s objective was to provide a starting point for prompts to be used for soliciting full, flowing and natural responses from informants. The seventeen semi-structured in-depth open-ended key informant interviews were conducted face to face. Interviews were held in English, Portuguese or French, according to the preference of the key informant. The 17 key informant interviews were transcribed verbatim. Notes taken during the interviews were used to complement the transcriptions collected as the primary data source. A transcription file of approximately 96 000 words maintained in the original language was created.

Emphasis was placed on overall quality and depth of the interviews conducted, rather than quantity. We considered saturation had been reached during data collection when no new themes came up, in line with Saunders et al.’s model of “data saturation” [26]. A strength of our study was the format and conduct of the 17 key informant interviews. The open-ended semi-structured nature allowed respondents to speak for extended periods without interruption by the interviewer. The circumstance that they were conducted in the language of choice (English, French or Portuguese) of the interviewees allowed respondents to express themselves in their preferred language. The verbatim transcription was also an advantage, creating a complete transcription file of more than 96 000 words upon which to conduct thematic analysis. A final strength is the range of stakeholders interviewed and the variety of roles and functions they have played in the response to HIV/AIDS in the country, whether in the public, private or non-governmental organization (NGO) sectors.

### Data analysis

This study was exploratory, iterative and inductive. Manual thematic analysis was conducted on verbatim transcripts in MS Word. Thematic analysis [27] was used to qualitatively code data. Codes were organized and synthesized into themes and sub-themes. The transcripts were compiled into a single file and read over several times in their entirety prior to the start of manual coding. Coding and identification of themes and sub-themes were conducted, and a consensus-based reading frame of the transcript data was developed, and dissenting opinions were resolved in an iterative manner. The themes were developed and explained in narrative form. The analysis included a comparison of stakeholders’ perceptions according to their profile, for example national or international, civil society or government. Differences in perceptions were noted where they were most striking. Relevant supporting quotations were identified to contextually bring to life the themes and sub-themes. We present the results according to each of the four themes that emerged from the data analysis, with each theme comprised of several sub-themes. Direct quotations from interviews conducted in French or Portuguese have been translated into English. Quality control was ensured by returning to key informants and seeking clarifications in cases when the interviewer had doubts about the meaning or subtlety of a particular statement.

### Ethical considerations

The interviewer explained the purpose and methodology of the study to all key informants. Participation was voluntary, and incentives were not offered. The confidential and anonymized nature of the data collection was emphasized prior to the start of the interview. Informed consent was requested, and all key informants were asked to sign a written informed consent form and a study registration form. All respondents provided verbal consent for their interview to be digitally recorded. Ethical approval was sought and provided by the national health ethics committee of Guinea-Bissau.

## Results

Interviews lasted between 30 and 70 min (average 55 min) and were usually conducted in the informant’s workplace. Sixteen interviews were arranged in person in the capital city of Bissau, and one took place over the phone. As summarized in Table 1 below, four overarching themes emerged from the transcribed key informant interviews, as follows: (1) the sense of constantly starting over and beginning again; (2) the effects of instability at central level that ripple down the health pyramid; (3) the idea that instability renders more vulnerable those who are already vulnerable; and finally, (4) coping mechanisms which have sprung up that have facilitated resilience in the HIV/AIDS response.

**Table 1:** Summary of main themes, subject themes, and supporting evidence

Main theme	Subject themes	Supporting evidence
Constantly starting over	*Frequent rotation and turn-over of staff working in the HIV/AIDS response* *Lack of handover*	“Start over and begin again.” “Reinvent the wheel”, “we never learn.”
Effects of instability rippling from central level throughout the health pyramid	*Changes at central level leading to changes in regional health directors* *Effects on people living with HIV/AIDS* *Outward emigration of health personnel, including HIV/AIDS specialists and care providers*	“The farther you go from Bissau… you don’t have this political instability problem.” “The butterfly effect, it starts from the top and when flapping the wings it drags all that [down].”
Vulnerable populations becoming more vulnerable	*Breakdown of the Guinean family* *Extreme vulnerability of girls and women to HIV infection* *Social and economic conditions favouring spread of HIV*	“Any political crisis… means that those who are vulnerable become much more vulnerable.” “At this period it was quite obvious that who provides for the family was the young girls.”
Coping mechanisms	*Community-led response to HIV/AIDS was early and has filled gaps* *Decentralized approaches* *Technical staff who keep working despite challenges*	“That was how [the NGO] practically emerged as a pioneer in filling the gaps left by the Ministry in the national response.” “If you want to promote changes in Guinea-Bissau, the best level to invest on is at local level where you can force changes at upstream level.”

### Constantly starting over in the HIV response

A politicized public administration—in which the management of the national HIV/AIDS response is embedded—characterized by frequent changes inevitably creates a high level of turnover, often in a context with limited or no formal handover procedures. Respondents portrayed the effect as having to constantly “start over and begin again,” complaining that they always “reinvent the wheel” and that “we never learn”. A phenomenon that has been termed “stable political instability” [23] then creates a kind of institutional inertia due to the constant changes and need to begin again. For example, more effort is required on the part of implementers to brief the new authorities. One civil society respondent with long experience in the fight against HIV/AIDS in Guinea-Bissau expressed this frustration:

“I had like seven or eight Ministers of Health during the ten years when I was there. Every time there was a change in government, we start all over the program again… I remember that every time there was a new Minister of Health, I had to go to Ministry of Health to explain… So with instability, every three months there is a new Minister, and every three months, we are forced to explain again how the different programs work…”

### Frequent rotation and turn-over of staff working in the HIV/AIDS response

Informants said frequent movement of staff created problems in the national response coordination. A new authority was named and begins anew, and implementers were obliged to go to the field and inform the new responsible staff of their activities. This took time and energy until the new authorities could attain a proper understanding and appreciation of their work.

“You know that the boss won’t last long, it won’t last long, consequences [are] if you are director of services or doctors, I know that in three or four months, in fact other people come, they don’t care about the changes, the changes won’t last very long. How sad.”

Cycles of instability stifle innovation in all aspects of the HIV/AIDS response as new and better practices rarely become part of the organizational culture and exacerbate staff apathy. Respondents further characterized instability’s effects on the HIV/AIDS response as being chronic, complete and widespread with abrupt changes. They did not have a clear sense of how or when the instability might end. There was also a strong perception that the instability had increased since the coup d’état of April 2012, particularly at the leadership and management levels of the HIV/AIDS response. One respondent recalled working with three different health ministers from 2006 to 2012, but with six ministers in the six years since 2012.

“These are sudden changes… Most of the management problem is these constant changes. Minister changes. You could only change the minister, but when you change, why change everyone? Others want to change the cleaning lady, do you understand? They want to change everything, change everything.”

### Lack of handover

Informants described the lack of handover procedures in place for the management of clinical data or policy information critical for the management of the HIV/AIDS response. When changes occurred, the new person was left with little documentation. Informants described information as “staying in the head” of the departing staff, and the information then being lost. Participants described how this had consequences resulting in taking up to a year for new personnel to come up to speed on the key issues. Trained staff moved on, and their replacements then needed to be trained. The following comment reflects upon both the chronicity of handovers, as well as the nature and quality of replacements in a highly politicized environment.

“When there is a change, a search, a political person comes who does not understand anything. It has consequences down to the bottom. Why did this person come, he doesn’t know [the city of] Gabú, he doesn't know [the city of] Bafatá… He is five or six months old [in his job], the government falls and another one comes out, what do you know? When is it that he is going to get acquainted, to know how is this, that and the other, this is missing, when is it that he is going to know, they train a person and in one day, the whole structure falls, everything falls…”

### Effects of instability rippling from central level throughout the health pyramid adversely affect the HIV response

Several respondents conceptualized the effects of political instability as rippling throughout the health system and dragging with it adverse consequences for the HIV/AIDS response down each tier of the health pyramid. Three common sub-themes that emerged were: (1) the effects on the regional health directorates, most often manifested in frequent rotations in Regional Health Directors, who are responsible for health issues in the region, including HIV/AIDS; (2) the negative effects faced by people living with HIV/AIDS; and finally, (3) the consequences on the quality and quantity of human resources for health, including HIV/AIDS specialists and providers. All respondents identified power struggles based in the capital city of Bissau as the epicenter of the country’s political instability. A quote from one correspondent captures this widespread sentiment:

“If you ask me why, I always say that the problem of Guinea-Bissau is Bissau, it’s not Guinea-Bissau. The people in Bissau. The farther you go from Bissau, you have economic problems, you have development problems, but you don’t have this political instability problem.”

Respondents described in different ways the “cascade effects” throughout the health pyramid that result from political instability emanating from the capital city. This participant put forward this metaphor of the health pyramid and emphasized the rotative nature of change, suggesting negative consequences for the management of the HIV/AIDS response at each tier of the health system:

“If we consider the structure of the Ministry is a pyramid, it means that everything falls in cascade, falls in cascade means from one level to another level, what happens on the first level… will have cascading effects until we get there to the base…”

### Changes at central level lead to changes in regional health directors

The perception across respondents was that changes at central level can lead to changes at regional level—which can include local health officials and clinical providers in the regional HIV/AIDS response—but not systematically and not automatically. For example, Regional Health Directors may be rotated or replaced, but not always. Another respondent called them “butterfly effects” that trickle throughout the country, down the health pyramid, and affect all layers of the HIV/AIDS response:

“The butterfly effect, it starts from the top and when flapping the wings it drags all that [down]… with our ministers, when it changes every three months, the entire health system changes completely down to the bottom every three months, the administrators of the regions also change.”

Politically driven changes at central level can lead to changes in regional health staff, which can lead to loss of knowledge of contextual issues specific to that region’s HIV/AIDS epidemic. This respondent clarified that rotation among regional directors depends on the person in question and party politics, explaining:

“It is something that comes in cascade, because a regional director of health comes from a certain region, from a party, and was nominated by the party. If there is another post that is in the regional office, and there is another party militant, then he can then nominate this person [party militant] from the moment of the competence to nominate, so it comes from top to bottom, these changes are frequent, with instability it is frequent…”

### Effects on people living with HIV/AIDS

When queried about potential effects of political instability on people living with or affected by HIV/AIDS, respondents mentioned various consequences including stigmatization, loss to follow up, viral resistance, stock-outs of drugs, and overall loss of patient confidence in the health system. One respondent spoke about the reticence that marginalized populations face in an environment where health care providers frequently change:

“In front of a doctor, three months later a new doctor comes, he has to see a new doctor. But let’s imagine this person has HIV and is a sex worker. There is double-stigmatization. She has to inform or should inform the doctor that she is HIV-positive and a sex worker. If this doctor leaves, another one comes in, she has to once again share her status with the new doctor so this creates discomfort in people’s confidence in the system…”

One doctor explained that high loss-to-follow-up rates could be explained by the movement of health care staff in a context lacking a robust patient monitoring system or personnel handover system, noting the challenges of patient follow up for laboratory testing:

“If you did the viral load on my child, and you did PCR on my son, and [I am] waiting for the results to come here, to the laboratory, he was the contact person with the laboratory, which would later be transferred to Bissau, when I call, ‘no, no I’m not there anymore,’ it’s closed, automatically, everything is closed.”

The confidence of people living with HIV or more vulnerable to HIV infection is eroded when they anticipate that the health system will fail to meet their needs, thus delaying or deciding not to seek health care, as expressed by this respondent:

“If the system is not going to solve my problem, I will not go… health structures are weaker and weaker. Those structures became weaker and are unable to respond to users, patients do not manage to get a response, users lose confidence in health structures. The health structures lose confidence and that has costs, in terms of patients who become more vulnerable.”

### Outward emigration of health personnel, including HIV/AIDS specialists and care providers

Although most respondents focused on instability’s “butterfly effects” from the top of the health pyramid down to the bottom, the outward emigration of health personnel involved in the HIV response in search of better living and working conditions in other countries could also be conceptualized as a cascade effect outside of the health system itself, as explained by one respondent:

“The conflict can lead to instability, it has led to the exit of health professionals to Europe, to [other African countries] outside the country… At one point more than a dozen doctors went to Portugal because of the conflict, the situation. Others went, others withdrew, a large number never came back, they are gone.”

### Vulnerable populations becoming more vulnerable

Political impasses negatively affect the entire population, and vulnerable groups the most, as they become more vulnerable during crises. One respondent summarized it this way:

“Because the country is unstable and has a vulnerable population, any political crisis during which the partners must turn their back means that those who are vulnerable become much more vulnerable.”

The sub-themes revolved around the moral dilemma faced by the family, the social and economic challenges faced within the (extended) family, the bedrock of Guinean society, and the exposure of young girls to heightened levels of risk-taking, which renders them more vulnerable to HIV/AIDS. Human rights violations are common in a context of limited social and economic opportunities.

### Breakdown of the Guinean family

Many respondents described changing social mores provoked by chronic political instability. The position of men as primary wage-earners has been seriously eroded, and many households, especially in cities and towns, are female headed. In rural areas, the “family” is ageing as younger generations move to urban areas, continuing a process of slow socio-economic erosion in place for decades. What interviewees put forward is an intra-household moral crisis within the nuclear family, exacerbated over time by socio-economic factors, increasing individual and collective vulnerability, and thereby negatively impacting risks related to HIV/AIDS. One respondent saw the negative effects of instability most acutely on the family:

“All these changes have ended up affecting the family… You see that there are parents who cannot guide their children, the HIV/AIDS prevalence rate has grown in an abysmal way. This means that there is a lack of control, a lack of control in which the head of the family has no control over the family, because he does not have the financial capacity to provide for the family… So this political instability has its repercussions even for the last member of the Guinean social family, in my perspective.”

### Extreme vulnerability of girls and women to HIV infection

Nearly all respondents spoke of the gender implications of the effects of political instability, most notably on the heightened vulnerability of adolescent girls and young women. Themes were repeated around promiscuity, infidelity, gender-based violence, early marriage and female genital mutilation. The proliferation of intergenerational relationships whereby young women offer informal transactional sex to older men was also raised repeatedly:

“With instability, their parents cannot afford to take care of the basic needs of children, they cannot afford to go to school, they cannot afford to feed them. At this period it was quite obvious that who provides for the family was the young girls… Most of the parents were aware of that situation. So, the instability, the political instability, one of the impacts on economic and social level was there. It was there. And it was obvious for everyone.”

### Social and economic conditions favoring spread of HIV

Respondents reflected on both the breakdown of the family and the vulnerability of girls and young women as potential income-earners through transactional sex. The precarious situation of the household often obliges parents to maintain their silence:

“Many parents know. But they do it because there is nothing to eat. Because the father has not received his salary for three or four months and because the salary he receives does not provide for the house. He knows for example that taking out a lot of bananas [to be sold by the daughter] will come [back] with a lot of money… but when the amount of bananas does not match what the child brings back, it’s money that enters the home.”

These vulnerabilities, particularly for women and girls, were viewed by some respondents through the lens of human rights. Issues related to impunity and access to justice, social inequality and women’s rights created an environment that fuels HIV transmission:

“The violation of human rights in Guinea-Bissau particularly at downstream level is serious…. People are not aware of their rights. Especially women. Issues related with inheritance, issues related with land poverty, issues related to gender-based violence. The famous issue of female genital mutilation. These are things across human rights.”

### Coping mechanisms

When asked about approaches to cope with the HIV/AIDS epidemic given the country’s political instability, respondents often spoke about the importance of community-led, local and grassroots initiatives that have been created to complement or substitute inexistent public sector services. Donors tended to emphasize the importance of supporting decentralized projects for HIV/AIDS in a context where the state is often minimally represented or absent. Another important sub-theme that emerged was the sense of hope felt by the Bissau-Guinean people that someday the political situation will improve, provide better access to HIV/AIDS and health services more generally, and create the conditions for a better quality of life for all.

### Community-led response to HIV/AIDS was early and has filled gaps

From the beginning of the HIV response in Guinea-Bissau, civil society organizations took it upon themselves to try to fill gaps left by government. Key NGO service providers were among the first providers of prevention of mother to child transmission and ART services, as described by one respondent:

“That was how [the NGO] practically emerged as a pioneer in filling the gaps left by the Ministry in the national response. The institutional failures at that time also failed to capitalize on [the NGO’s] investments… seeing [the NGO] as a competitor rather than a partner, agreeing on the collaboration protocol took a long time… So it created a bit of rivalry and that is the perception that I had.”

### Decentralized approaches

Like respondents’ emphasis on the importance of grassroots initiatives led by NGOs, many remarked that the most effective HIV/AIDS interventions are implemented at lower levels in the health system, rather than at the central level:

“If you want to promote changes in Guinea-Bissau, the best level to invest on is at local level where you can force changes at upstream level. The demand at local level will force changes at central level. If we continue only to invest at central level, those resources will never get there. They will never reach the local level because the state is not there.”

### Technical staff who keep working despite challenges

The resilience of the HIV/AIDS response depends on the efforts of technical staff who continue to do their work on a day-to-day basis, despite enormous challenges. This view was expressed by many national respondents, often around the notion of hope that the situation would someday improve, as expressed by this former Ministry staff active in the early days of the country’s HIV/AIDS response:

“I remember two Ministers were appointed within a week, and I went there with a document, and then [this Minister] was withdrawn and a new one comes in, but us [technicians] and the WHO Representative, we were always there with the document in hand, to show, it’s necessary to do this, this this…”

## Discussion

Our study examined stakeholders’ perceptions of the effects of chronic political instability on the HIV/AIDS response in Guinea-Bissau during the period of 2000–2015. Based on thematic analysis of transcriptions generated from seventeen in-depth key informant interviews, our results indicate that the effects of political instability have been pervasive and have inflicted profound suffering on the Bissau-Guinean people. Political instability affects all sectors of society, including health, and stakeholders perceive that since the inception of this instability, there have been specific adverse effects on the country’s HIV/AIDS response that persist to the present day. The impacts felt in the HIV/AIDS response are perhaps more readily noticeable than in other areas because effects on the health sector hold life or death implications for the population. Stakeholders perceive a phenomenon of “stopping and starting” and having to “restart at zero” in the HIV/AIDS response because of recurrent political changes and high rotation of personnel in key positions. These effects cascade down the health pyramid from central level to the most decentralized levels and affect all layers of the national HIV/AIDS response. Over time, the “stopping and starting” and “cascade effects” have rendered the most vulnerable to HIV/AIDS even more vulnerable, and to respond, early grassroots initiatives through communities and NGOs were organized to provide basic services and meet communities’ needs for HIV/AIDS prevention, treatment, care and support.

Our results are important because they raise new questions about the HIV/AIDS epidemic in Guinea-Bissau. The vulnerability of adolescent girls and young women in a context of extreme poverty and few income-generating opportunities contextualizes the feminization of the country’s HIV/AIDS epidemic. Prevalence of HIV-1/HIV-2 in 2016 was estimated to be more than three times higher among young women (ages 15–44 years) compared to young men in the same group (5.2% versus 1.7%) [4]. Our analysis of stakeholders’ perceptions of the effects of instability on the HIV/AIDS response are significant because the country’s political instability has not ended. Although our study had been designed to examine chronic political instability specifically during the Millennium Development Goals era (2000–2015), during the interviews, what became apparent was the difficulty for interviewees to differentiate the effects of political instability during this period compared to the post-2015 period. Guinea-Bissau’s instability is a phenomenon that continues to the present day [28], while trends in the country’s HIV/AIDS indicators do not appear to be improving, according to the limited impact and outcome data available.

The effects of political instability on Guinea-Bissau’s HIV/AIDS response, and more broadly on the entire health system, seem to be well-acknowledged by local stakeholders and discussed openly. Nevertheless, the candor with which key respondents spoke at length on the topic was surprising. Often requiring little prompting, respondents were able to pinpoint specific examples of its negative effects on the HIV/AIDS response. One tension highlighted in the responses relates to the effects of the high rotation of Ministers of Health. While the frequency of ministerial changes almost always brings negative consequences for planning and administration of the HIV/AIDS response, some respondents described that sometimes the changes are in fact positive, when a knowledgeable and committed Minister replaces one who is less capable or committed.

Other studies from Guinea-Bissau based upon interviews with key actors in and out the health sector likewise suggest a consensus that the 1998–1999 civil war and its aftermath, manifested in weak governance and pervasive corruption, have led to resentment and seriously compromised the implementation of social services, including health [29]. Despite these setbacks, interviewees also recognized the resilience associated with coping strategies, particularly locally led community-based approaches for addressing HIV/AIDS-related vulnerabilities, which aimed to shield implementation aspects from the negative impact of unstable governance [30]. The high levels of self-reliance, community-level resilience, and individuals’ hope in a better future is key to understanding Bissau-Guineans’ local coping mechanisms at individual, family, and community level.

Our findings are consistent with other research findings from similar countries or contexts which are experiencing prolonged periods of chronic political instability. In fact, there is significant research in medical anthropology and related disciplines about the relationship between political instability and health, including HIV/AIDS, and much of this work has been completed in sub-Saharan Africa and in other regions that experience political instability [31–34]. With respect to HIV/AIDS research on Guinea-Bissau, the literature has been largely biomedical in approach and output, and to our knowledge, this is the first attempt to look qualitatively at the forms and effects of political instability on the HIV/AIDS response in Guinea-Bissau. The use of qualitative research methods to investigate how people perceive phenomena can help to provide a starting point to define new and relevant research questions. Cookey et al. [35], referencing Bagheri et al. [36], recalls that “perception refers to the personal understanding of the phenomenon, causes and its effects, which influences necessary actions to be taken by the individual, group or community.” Understanding local perceptions of how policymakers and stakeholders experience the phenomenon of chronic political instability and the unique challenges it presents for national HIV/AIDS responses could lead to more and better evidence-based HIV/AIDS research and programming.

Our results may be generalizable to other contexts, including other former Portuguese colonies in Africa affected by instability and conflict. In what they term the “NGO-ization of community healthcare in Guinea-Bissau,” Baldursóttir et al. [37] has found results consistent with ours with respect to donor engagement directly with NGOs rather than government, perhaps more prevalent since the last military coup in 2012. In Mozambique, the theme of donor dependency in low-income settings has been explored by Høg [37] who notes the challenge of achieving national ownership in a crowded context of aid actors in the HIV/AIDS response, and by Mussa et al. [38] who describes inequalities created by disease-specific funding.

A potential limitation could be that the views cannot be considered representative of all stakeholders in the HIV/AIDS response. It has been noted previously in this journal that “studies based on perceptions… are likely to suffer from exaggeration or underestimation of issues” [39]. A potential limitation may have been the interviewer’s affiliation with the Global Fund to Fight AIDS, Tuberculosis and Malaria, in the role of the country’s Fund Portfolio Manager; however, it did not prevent respondents from sharing critical observations with the interviewer. The key informants all had pre-existing trust-based relationships with the interviewer. In most cases, this trust had been built over several years of collaboration on the HIV/AIDS response in Guinea-Bissau. Although this may have introduced bias, it likely enabled more authentic responses and insider views. Respondents may have reported information in line with social desirability bias due to the pre-established rapport; however, it is to be noted that findings are based on emerging themes with an aim of canceling out individuals’ biases. Finally, one could also point to the fact that the study did not include patients or service beneficiaries as key informants.

Further research may serve to provide a basis for recommendations for how national HIV/AIDS responses can be strengthened in non-conflict, low-resource contexts mired in the institutional inertia of political instability. More systematic inquiries into the phenomena of chronic political instability’s effects on HIV/AIDS outcomes would also be beneficial for future inquiry. With respect to the need to better understand the direct links between political instability and indicators that monitor HIV/AIDS programs, future epidemiological research in Guinea-Bissau should use quantitative measures to assess empirically the consequences of chronic political instability on the HIV/AIDS epidemic, as Rasmussen et al. have recently done [22]. More work should also be done to investigate the gender-related drivers and modes of HIV transmission in Guinea-Bissau, looking specifically at the vulnerabilities of adolescent girls and young women. Finally, our findings underline the importance of much needed reforms to strengthen the health sector by boosting the skills of a technocratic cadre within the Ministry of Health which should remain effectively shielded from political changes.

## Conclusions

Political instability can negatively affect health system organization and performance. Over recent decades, political instability has been manifested in Guinea-Bissau through a cycle of attempted and successful *coups d’état*, political assassinations, and the highly politicized roles of its armed forces. These events have had pernicious effects on the country’s fight against HIV/AIDS and could be one of the driving factors of its higher HIV/AIDS burden compared to its neighbors. Stakeholders from government, civil society, and donor organizations have recognized instability’s effects as a barrier to mounting an effective local response to HIV/AIDS in Guinea-Bissau.

Our study found that respondents were eager to share their perceptions of the effects that the country’s ongoing instability has, and continues to have, on the HIV/AIDS response. Respondents perceived they were obliged to constantly start over from zero, creating “butterfly effects” that descend from central level throughout the health system and negatively affect each layer of the HIV/AIDS response. Those vulnerable to HIV/AIDS are rendered more vulnerable, and as a result, local grassroots efforts have sprung up since the early stage of the epidemic to fill gaps in the HIV/AIDS response. Recognition of the heavy toll that political instability has taken on the country’s HIV/AIDS response is critical to any attempt to analyze and try to understand the country’s disease profile. To mitigate the effects of the country’s political instability on the HIV/AIDS response, and more generally on the entire health sector, concerted efforts should be made to strengthen the capacities of health officials within the Ministry of Health to shield them from the effects of the country’s political instability.

## Data Availability

The datasets generated and/or analyzed during the current study are not publicly available due to the risk of individual privacy of respondents becoming compromised.

## References

[1] World Bank. Guinea-Bissau Country Economic Memorandum: Terra Ranca! A Fresh Start. 2015. http://documents.worldbank.org/curated/en/347181468036532328/pdf/582960CEM0v20G0C0disclosed020250150.pdf. Accessed 14 April 2021.

[2] Forrest J (2003). Lineages of state fragility: rural civil society in Guinea-Bissau.

[3] Dabo, A. Guinea-Bissau crisis deepens with two rival presidents and prime ministers. Reuters. 2020. https://www.reuters.com/article/us-bissau-election/guinea-bissau-crisis-deepens-with-two-rival-presidents-and-prime-ministers-idUSKBN20N0PC. Accessed 14 April 2021.

[4] Olesen J, Jespersen S, da Silva ZJ, Rodrigues A, Erikstrup C, Aaby P, Wesje C, Hønge BL (2018). HIV-2 continues to decrease, whereas HIV-2 is stabilizing in Guinea-Bissau. AIDS.

[5] UNAIDS Data 2019. 2019. https://www.unaids.org/sites/default/files/media_asset/2019-UNAIDS-data_en.pdf. Accessed 14 April 2021.

[6] UNAIDS Country Fact Sheets: Guinea-Bissau 2018. 2018. https://www.unaids.org/en/regionscountries/countries/guinea-bissau. Accessed 15 December 2020.

[7] Stop TB. High Burden Countries. http://www.stoptb.org/countries/tbdata.asp. Accessed 14 April 2021.

[8] Ndokang EL, Tsambou AD (2015). Political instability in Central African Republic (CAR) and health state of the Cameroon population. J Life Eco.

[9] Kooiman J (1999). Social-political governance. Public Manage Int J Res Theory.

[10] Lewis M, Pettersson G. Governance in health care delivery: Raising performance. Policy Research Working Paper 5074. World Bank. 2009. http://documents.worldbank.org/curated/en/792741468330936271/pdf/WPS5074.pdf. Accessed 14 April 2021.

[11] World Health Organization. Health systems: Governance. 2020. https://www.who.int/healthsystems/topics/stewardship/en/. Accessed 14 April 2021.

[12] African Union. Goals and Priority Areas of Agenda 2063. 2020. https://au.int/agenda2063/goals. Accessed 14 April 2021.

[13] United Nations. World Population Prospects 2019: Data Booklet*.* 2019. https://population.un.org/wpp/Publications/Files/WPP2019_DataBooklet.pdf. Accessed 14 April 2021.

[14] Havik P, Chabal P, Green T (2016). Guinea-Bissau’s rural economy and society: a reassessment of colonial and post-colonial dynamics. Guinea-Bissau: microstate to narco-state.

[15] Central Intelligence Agency. The World Factbook: Guinea-Bissau. 2020. https://www.cia.gov/the-world-factbook/countries/guinea-bissau/. Accessed 14 April 2021.

[16] Ceesay H, Chabal P, Green T (2016). Guinea-Bissau: the ‘narco-state’ and the impact on institutions in Guinea-Bissau and countries in the sub-region. Guinea-Bissau: Microstate to narco-state.

[17] Forrest JB, Chabal P, Green T (2016). Guinea-Bissau’s colonial and post-colonial political institutions. Guinea-Bissau: Microstate to narco-state.

[18] Guinea-Bissau: Service Delivery Indicators Report—Health. World Bank. 2019. https://openknowledge.worldbank.org/handle/10986/32029. Accessed 14 April 2021.

[19] Kitson, N. Informal payments in the public health sector in Guinea-Bissau—Working Paper 564. Overseas Development Institute. 2019. https://cdn.odi.org/media/documents/12949.pdf. Accessed 14 April 2021.

[20] Månsson F, Biague A, da Silva ZJ, Dias F, Nilsson LAF (2009). Prevalance and incidence of HIV-2 and HIV-2 before, during and after a civil war in an occupational cohort in Guinea-Bissau. West Africa AIDS.

[21] Månsson F, Alves A, da Silva ZJ, Dias F, Andersson S, Biberfeld G (2007). Trends of HIV-1 and HIV-2 prevalence among pregnant women in Guinea-Bissau, West Africa: possible effect of the civil war 1998–1999. Sex Transm Infect.

[22] Gustafson P, Gomes V, Vieira C, Jensen H, Seng R, Norberg R (2001). Tuberculosis mortality during a civil war in Guinea-Bissau. JAMA.

[23] Russo G, Pavignani E, Guerreiro C, Neves C (2017). Can we halt health workforce deterioration in failed states? Insights from Guinea-Bissau on the nature, persistence and evolution of its HRH crisis. Hum Resour Health.

[24] Kok M, Rodrigues A, Silva A, de Haan S (2012). The emergence and current performance of a health research system: lessons from Guinea Bissau. Health Res Policy Syst.

[25] Rasmussen DN, Unger HW, Bjerregaard-Andersen M, Té DS, Vieira N, Oliveira I (2018). Political instability and supply-side barriers undermine the potential for high participation in HIV testing for the prevention of mother-to child transmission in Guinea-Bissau: a retrospective cross-sectional study. PLoS ONE.

[26] Saunders B, Sim J, Kingstone T, Baker S, Waterfield J, Bartlam B (2018). Saturation in qualitative research: exploring its conceptualization and operationalization. Qual Quant.

[27] Braun V, Clarke V (2006). Using thematic analysis in psychology. Qualitat Res Psychol.

[28] Institute for Security Studies. Stand-off following presidential elections in Guinea-Bissau. Peace and Security Council Report. 2020. https://issafrica.org/pscreport/psc-insights/stand-off-following-presidential-elections-in-guinea-bissau. Accessed 14 April 2021.

[29] Voz di Paz and Interpeace. Roots of conflicts in Guinea-Bissau: the voice of the people. 2010. http://www.interpeace.org/wpcontent/uploads/2010/08/2010_GB_Interpeace_Voz_Di_Paz_The_Voice_Of_The_People_EN.pdf. Accessed 14 April 2021.

[30] Guerreiro C, Hartz Z, Ferrinho P, Havik PJ (2019). 25 Anos de Política Nacional de Saúde na República da Guiné-Bissau; Memórias do seu Planeamento Estratégico em Saude. Cadernos de Estudos Africanos.

[31] Benton A (2015). HIV exceptionalism: development through disease in Sierra Leone.

[32] Crane J (2013). Scrambling for Africa: AIDS, expertise, and the rise of American global health science.

[33] Nguyen V (2010). The republic of therapy: triage and sovereignty in West Africa’s time of AIDS.

[34] Farmer P (2001). Infections and inequalities: the modern plagues.

[35] Cookey PE, Darnswasdi R, Ratanachi C (2016). Local people’s perceptions of Lake Basin water governance performance in Thailand. Ocean Coast Manage.

[36] Bagheri A, Shabanali Fami H, Rezvanfar A, Asadi A, Yazdani S (2008). Perceptions of paddy farmers towards sustainable agricultural technologies: case of Haraz catchments area in Mazandaran province of Iran. Am J Appl Sci.

[37] Baldursdóttir S, Gunnlaugsson G, Einarsdóttir J (2018). Donor dilemmas in a fragile state: NGO-ization of community healthcare in Guinea-Bissau. Dev Stud Res.

[38] Høg E (2014). HIV scale-up in Mozambique: exceptionalism, normalisation and global health. Glob Public Health.

[39] Mussa A, Pfeiffer J, Gloyd S, Sherr K (2013). Vertical funding, non-governmental organizations, and health system strengthening: perspectives of public sector health workers in Mozambique. Hum Resour Health.

[40] Amo-Adjei J (2013). Views of health service providers on obstacles to tuberculosis control in Ghana. Infect Dis Poverty.

